# Effect of Pediatric Rehabilitation on Children With Attention Deficit Hyperactivity Disorder (ADHD): A Case Report

**DOI:** 10.7759/cureus.62739

**Published:** 2024-06-20

**Authors:** Sakshi Desai, H V Sharath, Moh'd Irshad Qureshi, Raghumahanti Raghuveer

**Affiliations:** 1 Department of Pediatric Physiotherapy, Ravi Nair Physiotherapy College, Datta Meghe Institute of Higher Education and Research, Wardha, IND; 2 Department of Neuro-Physiotherapy, Ravi Nair Physiotherapy College, Datta Meghe Institute of Higher Education and Research, Wardha, IND

**Keywords:** pediatric physiotherapy, sensory integration therapy, adhd, rehabilitation, pediatrics

## Abstract

This case study examines a three-year-old male child with attention deficit hyperactivity disorder (ADHD) who exhibits fine motor impairments, language and speech delays, and delayed social milestones. The therapeutic intervention included a comprehensive program involving parent education, parental behavioral therapy, sensory integration therapy, treadmill walking, music therapy, and the Picture Exchange Communication System (PECS). The results showed significant improvements in the child's functional independence, behavioral management, and communication abilities, highlighting the efficacy of the multifaceted physiotherapy approach.

## Introduction

Among the most prevalent developmental disorders, attention deficit hyperactivity disorder (ADHD) often continues into adulthood [1]. ADHD affects between 3% and 7% of the global population, with 5% of children and 4% of adults displaying symptoms [2]. The primary symptoms of ADHD include hyperactivity, difficulty maintaining focus, impulsive behavior, and challenges adhering to plans and goals. Due to its high prevalence, ADHD can negatively impact social relationships, academic performance, overall well-being, and the development of social skills [3,4].

Research has consistently identified deficits in executive functions as a key characteristic of ADHD, particularly in response inhibition, attention, and working memory. Individuals with ADHD often continue to exhibit significant symptoms into adulthood, placing them at a higher risk for adverse long-term outcomes, such as lower educational and employment achievements, substance abuse, and adult psychiatric disorders, compared to their non-ADHD counterparts [5-9].

ADHD is defined by three main behavioral symptoms: (i) inattention, (ii) hyperactivity, and (iii) impulsivity. This disorder greatly affects individuals due to impairments in cognitive functions (e.g., challenges with memory, learning, and focusing on new tasks) and motor functions (e.g., difficulties in performing simple tasks such as writing or engaging in complex activities like sports), which can disrupt their personal, social, and professional lives. Skills such as maintaining attention, minimizing distractions, and enhancing response control are especially crucial in these areas [10-12].

Pediatric rehabilitation plays a crucial role in the management of ADHD in young children, particularly for a three-year-old male child. Early intervention is essential to address developmental delays and impairments in fine motor skills, language, speech, and social interactions. Through tailored therapeutic strategies, pediatric rehabilitation can enhance functional abilities, promote independence, and improve the overall quality of life. By engaging the child in structured activities and involving parents through education and behavioral therapy, comprehensive rehabilitation fosters a supportive environment that encourages developmental progress and better long-term outcomes for children with ADHD.

## Case presentation

Prenatal history

Prior to the child's assessment and treatment, the mother provided her consent. In this case study, a three-year-old kid with attention deficit disorder and speech difficulties is examined. He was diagnosed with ADHD. The prenatal history suggests an uneventful pregnancy, with the woman attending prenatal visits on a regular basis and no major problems to report. Her health was stable throughout the pregnancy, and she did not have any long-term health issues. Prenatal screenings, such as genetic testing and ultrasounds, revealed no anomalies or indications of developmental problems in the developing fetus.

Natal history

The child was born at full term to a Gravida1 Pareity1 Living1 Abortion0 mother through normal vaginal delivery, weighing 2.9 kg at birth. The baby had a cephalic presentation, and there was no umbilical cord around the neck. The baby cried immediately after birth. The APGAR scores were recorded as 9/10 at one minute and 9/10 at five minutes. The newborn showed proper feeding habits and physiological responses.

Presentation and diagnosis

According to the primary caregiver (mother), the child exhibited normal development up to the age of two, and the parents did not notice any signs of hyperactivity. During infancy, the child fell from the bed twice. The parents took him to the doctor, but there was no major trauma, and the doctor prescribed medication. As the child grew older, neighbors noticed behavioral changes and informed the parents. After the neighbors' observations, the parents began to monitor the child more closely and eventually took him to a local doctor, who diagnosed the child as hyperactive. Following the diagnosis, the doctor referred the child to the Department of Pediatric Physiotherapy at Acharya Vinoba Bhave Rural Hospital in Sawangi (Meghe), India, for the treatment of hyperactivity and attention deficit. The assessment of the child was done, and the treatment was initiated.

Observational findings revealed that the child does not remain seated in one place, has limited and unclear speech, and is constantly on the move. He exhibits a tendency to explore his environment continuously. The child tends to cry upon entering the Pediatric OPD and only engages with toys for a short period. His play behavior is not age-appropriate, and he shows minimal interaction with therapists and other children in the OPD. He demonstrates poor sustainability in activities and does not respond to his name or make eye contact. Additionally, imitation is absent, and he does not follow commands. The clinical examination of anthropometric findings is adequate for the child's age and is presented in Table 1.

**Table 1:** Clinical examination of anthropometric measurements at birth and present, showing changes in weight, height, chest circumference, and head circumference.

Anthropometric Measurement	At Birth	At Present (At the Age of 3 Years)
Weight	2.9 kg	14 kg
Height	44 cm	102 cm
Chest circumference	32 cm	53 cm
Head circumference	33 cm	50 cm

The developmental milestones related to gross motor skills were normal. A comprehensive summary of gross motor development is presented in Table 2.

**Table 2:** Clinical examination of gross motor milestones, detailing the expected and actual ages at which various gross motor skills were attained.

Gross Motor	Normal	Attained Age
Head control	6 weeks	3 months
Rolling	4-6 months	6 months
Sitting	5-7 months	7 months
Creeping	6-8 months	8 months
Crawling	9-11 months	9 months
Standing with support	9-12 months	10 months
Walking with support	10-15 months	12 months
Standing without support	11-13 months	13 months
Walking without support	13-16 months	15 months
Climbing stairs with support	16-18 months	17 months
Jumping	28-30 months	28 months
Climbing stairs without support	30-36 months	31 months

The child's fine motor milestones were delayed. A comprehensive summary of fine motor development is presented in Table 3.

**Table 3:** Clinical examination of fine motor milestones, detailing the expected and actual ages at which various fine motor skills were attained.

Fine Motor Milestones	Normal	Attained Month
Grasp reflex	0-3 months	5 months
Reach		6 months
Release	3-6 months	9 months
Mouthing	3-6 months	10 months
Transfers	4-6 months	16 months
Grasp	6-8 months	18 months
Scribbling	12-14 months	2 years
Stacking	14-17 months	Not achieved
Manipulations	28-30 months	Not achieved
Cribbling	32-36 months	Not achieved

Table 4 provides a clinical examination of language milestones, highlighting the expected and actual ages at which various language skills were attained. The patient turned his head to sound at six months, cooed at seven months, produced monosyllables at eight months, and disyllables by one year. Two-word phrases with meaning were spoken at two years, and 10 words with meaning were achieved by 2.5 years. However, the milestones of forming simple sentences and telling a story, expected by 24 months and 36 months, respectively, have not been attained.

**Table 4:** Clinical examination of language milestones, detailing the expected and actual ages at which various language milestones were attained.

Language Milestones	Normal	Attained Month
Turns head to sound	6 weeks	6 months
Cooing	3 months	7 months
Monosyllables	6 months	8 months
Disyllables	9 months	1 year
Two words with meaning	12 months	2 years
10 words with meaning	18 months	2.5 years
Simple sentence	24 months	Not attained
Telling a story	36 months	Not attained

Table 5 outlines the personal and social milestones, showing the expected and actual ages of achievement. The child exhibited a social smile at 1.3 years, recognized his mother at four months, smiled at the mirror by 2.5 years, and waved bye-bye at 1.5 years. They played a simple ball game at one year, but the milestone of knowing their gender, expected by 36 months, has not been attained.

**Table 5:** Clinical examination of personal and social milestones, detailing the expected and actual ages at which various personal and social milestones were attained.

Personal and Social Milestones	Normal	Attained Month
Social smile	1 month	1.3 years
Recognizing mother	3 months	4 months
Smiles at the mirror	6 months	2.5 years
Waves bye-bye	9 months	1.5 years
Plays a simple ball game	12 months	1 year
Knows gender	36 months	Not attained

Table 6 details the patient's activities of daily living, indicating levels of dependence. The patient is dependent on his mother for brushing his teeth, bathing, and toileting, needing assistance with washing and wearing a diaper when away from home. He is partially dependent on dressing, requiring help with buttons and zippers. He eats independently, although his hands get messy. He is independent in ambulation and transfers.

**Table 6:** Clinical examination of daily living activities, illustrating the patient's level of independence or dependence on these tasks.

Activities of Daily Living	Level of Dependency	
Brushing	Dependent	He is dependent on his mother for brushing his teeth. His mother makes him brush his teeth.
Bathing	Dependent	His mother bathes him; he can apply soap with her assistance and pour water on himself.
Toileting	Dependent	He is dependent on his mother to wash himself after using the toilet. He wears diapers when he is away from home.
Dressing	Partially dependent	He is partially dependent on his mother for dressing. He needs help with buttoning and zipping his clothes.
Eating	Independent	He is independent in eating, but his hands get messy.
Ambulation	Independent	-

Table 7 represents a tailored rehabilitation protocol that included parent education, parental behavioral therapy, sensory integration therapy, treadmill walking, music therapy, and Picture Exchange Communication System (PECS). The rehabilitation program lasted for six months, consisting of daily one-hour sessions. The primary caregiver was informed of the strategies to be implemented at home.

**Table 7:** Physiotherapy intervention.

Intervention	Procedure	Rationale
Sensory integration therapy [13]	Pegboard activity in a closed environment. The child sits with the therapist in a dimly lit room, where only minimal light is present. The therapist gives auditory commands, guiding the child to place the pegs into specific holes.	Engaging in pegboard activities requires precise hand-eye coordination and manipulation of objects, which helps improve fine motor activity. As the child works on placing pegs into specific holes, they practice focusing their attention on one specific task at hand, which helps in improving attention span over time. Arranging pegs in specific patterns helps improve visual-spatial skills and spatial awareness and organization. Pegboard also helps in calming and grounding tasks. The repetitive nature of the activity and the feedback of successfully placing pegs can help promote self-regulation and emotional stability.
	Clay Therapy introduces specific clay activities to the child; the activities can include rolling, kneading, pinching the clay, or sculpting specific objects.	Manipulating clay provides tactile sensory input, which helps in regulating sensory processing in the child. The child needs to focus on shaping and molding the clay to create specific forms or structures, which helps improve attention span. Clay therapy provides a non-verbal outlet for children, manipulating clay to externalize and process their emotions, reducing stress, and promoting emotional regulation. Kneading, rolling, pinching, and shaping clay helps develop and strengthen fine motor activity. It improves hand-eye coordination and dexterity, which is important for tasks such as writing and drawing.
	Scribbling, the child sits on the CP chair. With the help of a pencil, the child is asked to scribble on the paper. The therapist prompts and provides auditory cues such as “Can you draw a big circle” or “What colors do you want to use”. While the child is scribbling, the therapist introduces words like “apple” and “ball” to the child.	Scribbling provides sensory stimulation through the tactile experience of holding and manipulating the pen or pencil and the visual feedback of seeing the marks appear on paper. The sensory input helps regulate arousal levels and promote sensory integration. It improves attentional span and serves as a calming and soothing activity that promotes relaxation and emotional regulation. It helps improve fine motor activity.
	In treadmill walking [14], the child walks on a treadmill set to a comfortable speed. The therapist stands behind the child, providing auditory cues.	Treadmill walking provides a controlled environment for aerobic exercise, which helps in the release of excess energy and reduces hyperactivity. Treadmill walking requires sustained attention to maintain balance and coordination, which helps in improving concentration and attention over a span of time. Treadmill walking provides proprioceptive and vestibular input, which help in the regulation of arousal levels and promote a calmer state of mind. It also makes the child feel more organized and in control of their environment.
Music therapy [15]	The child experiences music that has been altered and adjusted, including different volume levels and pitches.	Engaging in music therapy requires sustained attention to rhythm, melody, and timing, which can improve attention span over time. Music can evoke emotions and serves as a tool for emotional expression and regulation. Music therapy provides sensory inputs that regulate arousal levels and promote sensory integration.
Picture Exchange Communication System (PECS) [16, 17]	The child is taught to initiate communication by exchanging a picture card of any desired item. The therapist promotes and gives reinforcement to encourage the child to make the exchange.	PECS will help children with auditory processing and attention, making it challenging for them to understand and remember verbal instructions or communications. PECS provides visual support through the use of pictures, which enhances comprehension and facilitates communication. It helps structured communication. It provides an alternative mode of communication. It encourages social interaction and engagement with peers and caregivers.
Parenteral behavioral training [18]	Parental behavioral therapy involves educating parents about the condition and effective parenting techniques, setting achievable goals, implementing positive reinforcement strategies, maintaining consistency in rules and consequences, effective communication, developing problem-solving skills, managing parental stress, promoting collaboration with other caregivers and professionals, regularly evaluating progress, and prioritizing parental self-care.	Parental behavioral therapy aims to equip parents with effective strategies to manage their child's symptoms by promoting positive reinforcement, consistency, and communication. This approach recognizes the critical role parents play in creating a supportive environment and aims to reduce parental stress while enhancing the child's overall well-being and behavior.

**Figure 1:**
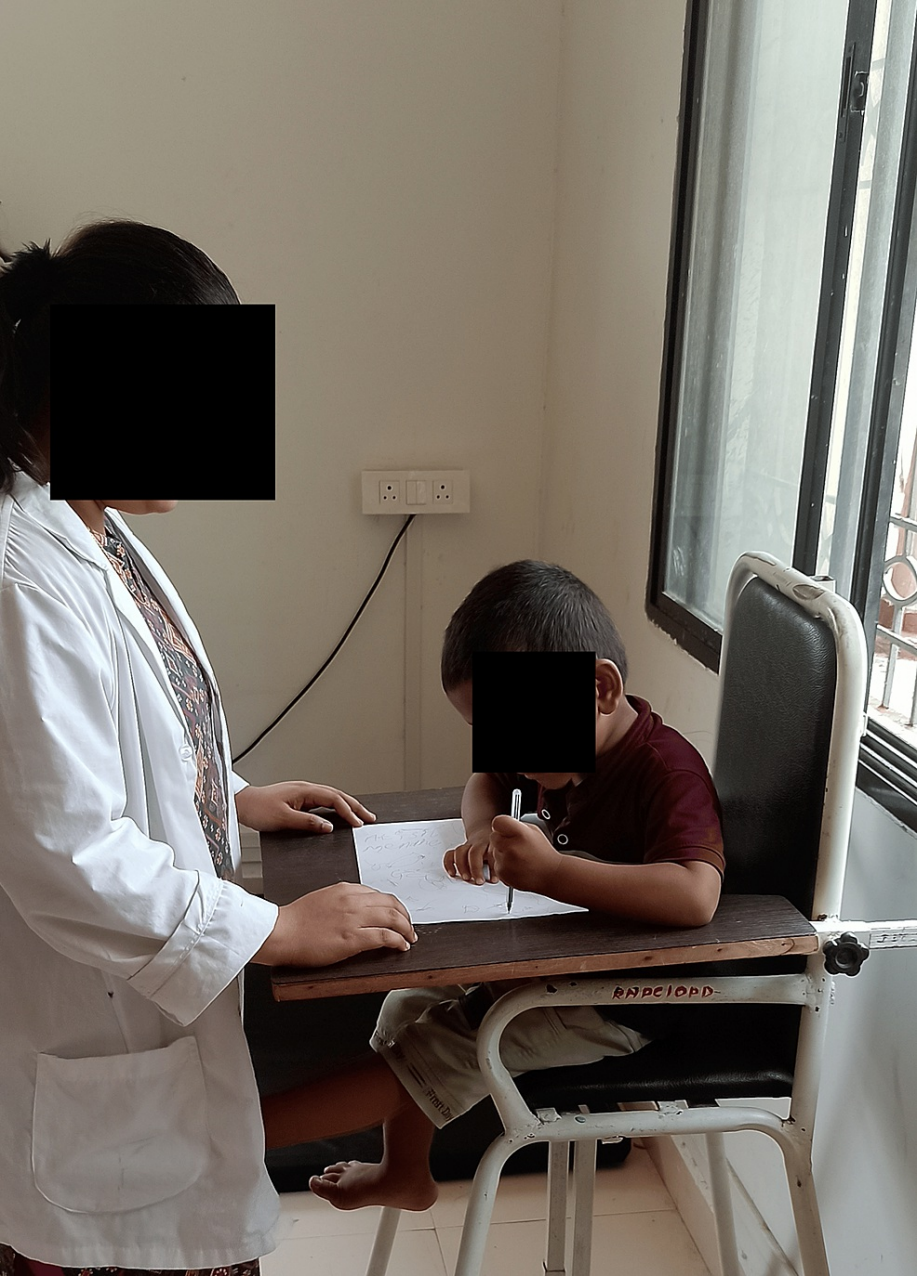
Scribbling.

The sensory integration therapy involves pegboard activities in a dimly lit room where the therapist gives auditory commands to guide the child in placing pegs, enhancing fine motor skills, attention span, visual-spatial skills, and promoting emotional stability. Clay therapy engages the child in rolling, kneading, and sculpting clay, providing tactile sensory input that improves attention span, fine motor skills, emotional regulation, and sensory processing. Scribbling involves the child sitting in a CP chair and using a pencil to draw while receiving auditory cues from the therapist, improving sensory stimulation, attention span, fine motor skills, and emotional regulation. Treadmill walking, with the therapist providing auditory cues, helps release excess energy, improves concentration and attention, and provides proprioceptive and vestibular input, promoting a calmer state of mind and a sense of control. Music therapy, through exposure to varied rhythms, melodies, and pitches, enhances attention span, emotional expression, and sensory integration. PECS teaches the child to initiate communication by exchanging picture cards, improving auditory processing, comprehension, structured communication, and social interaction. Parental behavioral training educates parents on effective parenting techniques, positive reinforcement, and stress management, fostering a supportive environment that enhances the child's behavior and overall well-being (Figure 1).

**Figure 2:**
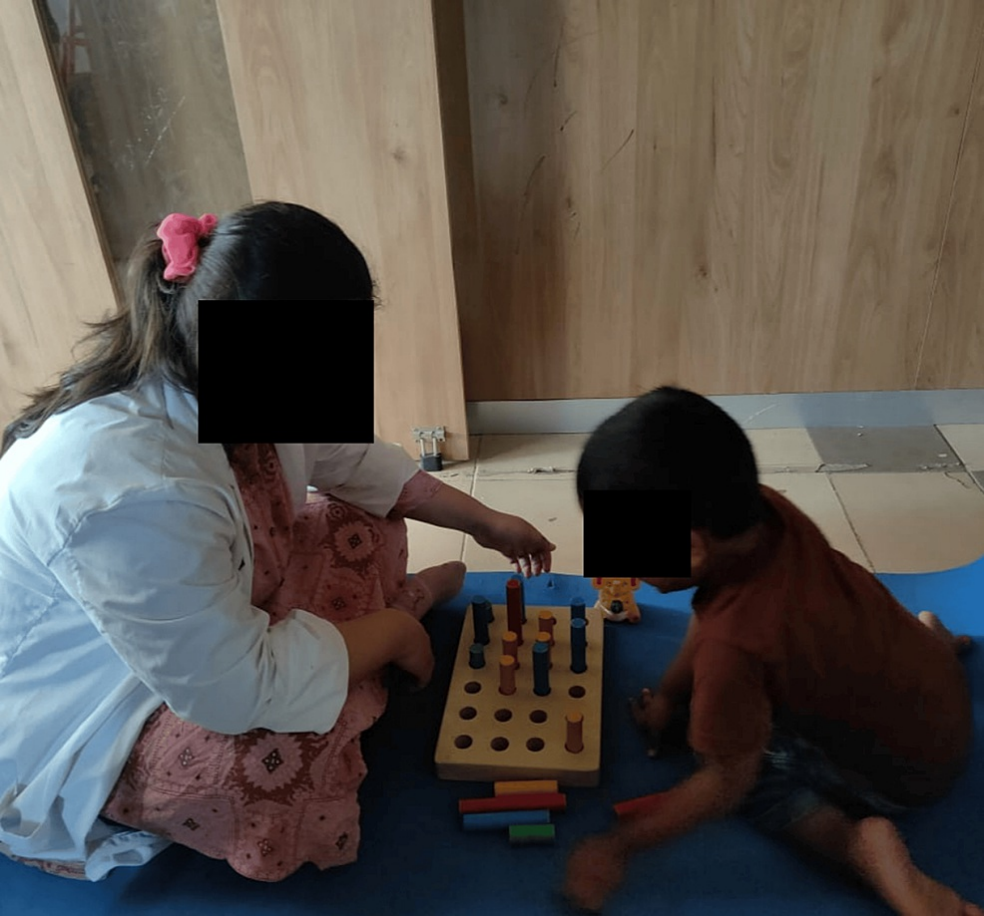
Pegboard activity in a dimly lit room.

The pegboard activity, performed in a dimly lit room, enhances sensory awareness and fine motor skills through a subtle interplay of light and shadow. As participants focus intently on placing pegs into holes, the low lighting conditions heighten their tactile sensitivity, requiring them to rely more on touch than sight. This environment fosters a deeper concentration, minimizing visual distractions and promoting a sense of calm and introspection. The gentle ambiance also creates an atmosphere conducive to mindfulness, allowing individuals to immerse themselves fully in the task at hand, ultimately leading to improved dexterity and hand-eye coordination (Figure 2).

**Figure 3:**
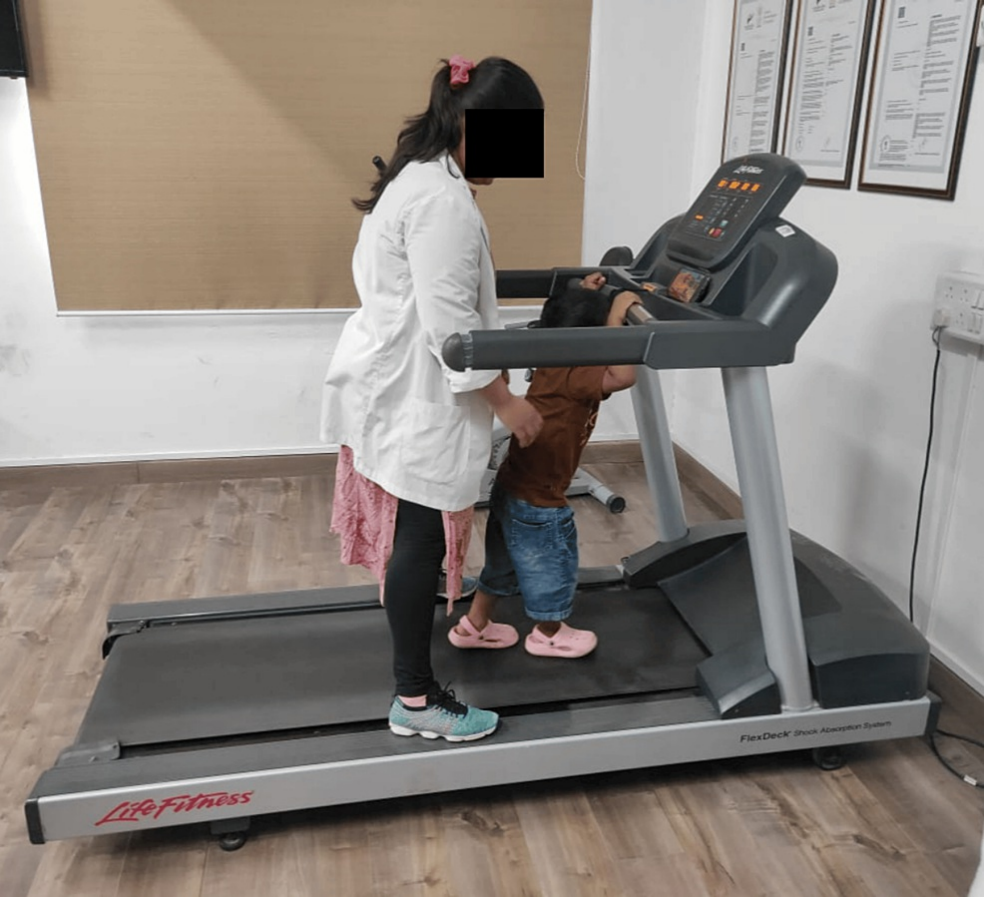
Treadmill walking.

Incorporating treadmill exercises into the routines of children with ADHD can be highly beneficial for managing symptoms and enhancing overall well-being. Physical activity, such as walking or running on a treadmill, has been shown to improve focus, reduce hyperactivity, and enhance mood by increasing the levels of neurotransmitters such as dopamine and norepinephrine in the brain. Structured treadmill sessions provide a controlled environment for children to expend excess energy, helping to mitigate restlessness and impulsivity. Additionally, regular aerobic exercise can enhance cognitive function, support better sleep patterns, and contribute to overall physical health, making it a valuable component of a comprehensive ADHD management plan (Figure 3).

**Figure 4:**
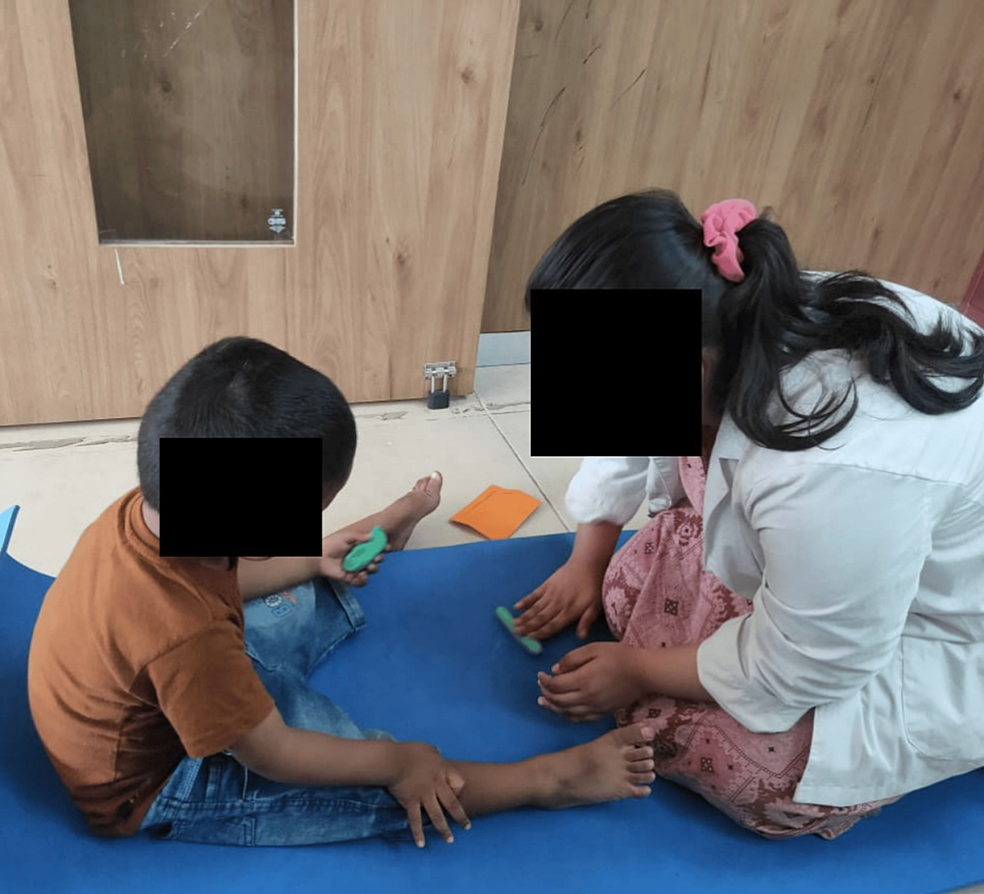
Clay therapy.

Clay therapy (Figure 4), also known as clay art therapy, is a form of expressive therapy that involves the use of clay as a medium for emotional expression and psychological healing. This tactile and hands-on approach allows individuals to mold, shape, and create objects, facilitating a connection between their inner experiences and external creations. The act of working with clay can be calming and grounding, helping to reduce anxiety and stress. For children, it enhances fine motor skills, encourages creativity, and provides a non-verbal outlet for emotions that might be difficult to articulate. For adults, clay therapy can uncover subconscious thoughts and feelings, promote self-discovery, and aid in processing traumatic experiences. Overall, clay therapy offers a unique and effective way to explore and heal emotional issues through artistic expression and sensory engagement.

Pre-physiotherapy, the child struggled with eye contact, social interaction, attention, speech, communication, emotional expression, play behavior, and obeying commands. Post-intervention, notable improvements were seen across all areas, with the child now confidently engaging with others, maintaining focus, communicating effectively, regulating emotions, and demonstrating age-appropriate play and obedience. A detailed summary is presented in Table 8.

**Table 8:** This table summarizes the changes observed in the child before and after physiotherapy intervention, highlighting improvements in various developmental and social aspects.

Category	Pre-Physiotherapy Intervention	Post-Physiotherapy Intervention
Eye contact	The child did not make eye contact.	The child makes eye contact.
Social smile	The social smile was not present.	The child smiles with family and peers around.
Attention	The child easily got distracted, struggled to stay focused on tasks, and was always on the go.	The child sustains attention, remains focused on the task, and organizes activities.
Speech	The child could not speak properly.	The child is able to speak and read the alphabet, numbers, and words.
Communication	The child did not talk with peers or the therapists.	The child talks and plays with the therapist and the peers and replies to questions.
Emotional regulation	The child did not express any emotions.	The child smiles, laughs, enjoys the play, and even cries.
Play behavior	The child was disorganized and not age-appropriate.	The child shows organized behavior and plays with simple puzzles.
Obeying commands	The child did not obey the commands given to him.	The child obeys the commands given to him.

## Discussion

This case study describes a three-year-old boy with ADHD who had attention and speech difficulties. Over six months of a structured pediatric rehabilitation program, he showed significant improvements in speech fluency and attention span. Sensory integration therapy, including activities such as pegboard tasks, clay play, and scribbling, provided tactile experiences that regulated sensory input, enhanced fine motor skills, and promoted emotional stability, helping him to focus better. Treadmill walking helped expend excess energy and provided calming sensory feedback, while music therapy assisted in managing emotions and improving sensory processing. The PECS structured his communication, reducing frustration and enhancing social engagement [19].

The physiotherapy intervention led to notable developmental progress. One significant improvement was the child’s ability to maintain eye contact, an essential aspect of social interaction. He also began smiling during interactions with family and peers, indicating better emotional responsiveness and social connection. His attention and focus greatly improved, enabling him to sustain attention, stay on task, and organize activities more effectively, reflecting better cognitive and executive functioning. His speech and language development advanced significantly as he learned to speak and read the alphabet, numbers, and words, showing remarkable language acquisition [20]. His communication skills improved substantially, allowing him to engage in conversations, play with therapists and peers, and respond to questions, marking a considerable enhancement in social interaction and language use. His emotional regulation also improved as he started expressing a wider range of emotions, such as smiling, laughing, enjoying play, and even crying, indicating healthier emotional development. His play behavior became more organized and age-appropriate, and he began to solve simple puzzles, demonstrating improved cognitive and motor skills. Additionally, he started obeying commands, reflecting better comprehension, attention, and behavior.

Overall, the outcomes six months after the intervention underscore the effectiveness of the physiotherapy program in addressing the child’s developmental delays and enhancing social, emotional, and cognitive functioning. The combination of therapies led to progressive improvements in attention span, communication abilities, fine motor skills, and overall behavior. Continuous monitoring and adjustments to the intervention plan are essential to meeting the child's evolving needs. Further research and case studies could provide additional validation for such comprehensive therapeutic approaches for children with ADHD.

## Conclusions

The case report on the effect of pediatric rehabilitation on a child with ADHD highlights the significant improvements observed through a structured rehabilitation program. The child demonstrated notable progress in attention span, impulse control, and overall behavioral management. This improvement was facilitated by a multidisciplinary approach involving occupational therapy, behavioral therapy, and parent training, which collectively addressed the core symptoms of ADHD. The tailored interventions were crucial in enhancing the child's academic performance and social interactions, underscoring the importance of individualized care in pediatric rehabilitation for ADHD.

In conclusion, this case report emphasizes the positive impact of comprehensive pediatric rehabilitation on children with ADHD. The integration of various therapeutic strategies not only mitigates the symptoms of ADHD but also promotes long-term developmental benefits. This case advocates for the adoption of a holistic and individualized treatment approach, encouraging further research and implementation in clinical practice to support children with ADHD in achieving their full potential.

## References

[1] Mahone EM, Denckla MB (2017). Attention-deficit/hyperactivity disorder: a historical neuropsychological perspective. J Int Neuropsychol Soc JINS.

[2] Ercan ES, Kandulu R, Uslu E (2013). Prevalence and diagnostic stability of ADHD and ODD in Turkish children: a 4-year longitudinal study. Child Adolesc Psychiatry Ment Health.

[3] Zoccolillo M (1993). Gender and the development of conduct disorder. Dev Psychopathol.

[4] Polanczyk G, de Lima M S, Horta B L, Joseph B, Luis Augusto R (2007). The worldwide prevalence of ADHD: a systematic review and metaregression analysis. Am J Psychiatry.

[5] Johnson S, Hollis C, Kochhar P, Wolke D, Marlow N (2010). Psychiatric disorders in extremely preterm children: longitudinal finding at age 11 years in the EPICure study. J Am Acad Child Adolesc Psych.

[6] Brogan E, Cragg L, Gilmore C, Marlow N, Simms V, Johnson S (2014). Inattention in very preterm children: implications for screening and detection. Arch Dis Child.

[7] Breeman LD, Jaekel J, Baumann N, Bartmann P, Wolke D (2016). Attention problems in very preterm children from childhood to adulthood: the Bavarian Longitudinal Study. J Child Psychol Psych.

[8] Scott MN, Taylor HG, Fristad MA, Klein N, Espy KA, Minich N, Hack M (2012). Behavior disorders in extremely preterm/extremely low birth weight children in kindergarten. J Dev Behav Pediatr.

[9] Nigg J T, Stavro G, Ettenhofer M, Miller T, Henderson JM (2005). Executive functions and ADHD in adults: evidence for selective effects on ADHD symptom domains. J. Abnorm Psychol.

[10] Seidman L J, Valera E M, Makris N, Aleradi M, Faraone S, Beiderman J (2006). Dorsolateral prefrontal and anterior cingulated cortex volumetric abnormalities in adults with attention-deficit/hyperactivity disorder identified by magnetic resonance imaging. Biol Psych.

[11] Ghanizadeh A (2011). Sensory processing problems in children with ADHD, a systematic review. Psychiatry Investig.

[12] Durgut E, Orengul AC, Algun ZC (2020). Comparison of the effects of treadmill and vibration training in children with attention deficit hyperactivity disorder: a randomized controlled trial. Neuro Rehab.

[13] Park JI, Lee IH, Lee SJ, Kwon RW, Choo EA, Nam HW, Lee JB (2023). Effects of music therapy as an alternative treatment on depression in children and adolescents with ADHD by activating serotonin and improving stress coping ability. BMC Complement Med Ther.

[14] Zhu C (2022). Effects of musicotherapy combined with cognitive behavioral intervention on the cognitive ability of children with attention deficit hyperactivity disorder. Psychiatr Danub.

[15] Kolhe PD, Sharath HV, Thakre VM, Ankar P (2023). Multimodal physiotherapy approach for autism with speech impairment and attention deficit: a case report. Cureus.

[16] Sciberras E, Efron D, Patel P (2019). Does the treatment of anxiety in children with attention-deficit/hyperactivity disorder (ADHD) using cognitive behavioral therapy improve child and family outcomes? Protocol for a randomized controlled trial. BMC Psychiatry.

[17] Sagvolden T, Johansen EB, Aase H, Russell VA (2005). A dynamic developmental theory of attention-deficit/hyperactivity disorder (ADHD) predominantly hyperactive/impulsive and combined subtypes. Behav Brain Sci.

[18] Srushti Sudhir C, Sharath HV (2023). A brief overview of recent pediatric physical therapy practices and their importance. Cureus.

[19] Willcutt EG, Nigg JT, Pennington BF (2012). Validity of DSM-IV attention deficit/hyperactivity disorder symptom dimensions and subtypes. J Abnorm Psychol.

[20] Posner J, Polanczyk GV, Sonuga-Barke E (2020). Attention-deficit hyperactivity disorder. Lancet.

